# Supplementary material for: Causal Relationship between Aging and Anorexia Nervosa: A White-Matter-Microstructure-Mediated Mendelian Randomization Analysis
Source: Biomedicines. 2024 Aug 16;12(8):1874. doi: 10.3390/biomedicines12081874 (PMC11351342; doi:10.3390/biomedicines12081874)
Supplement: Supplementary file 1 [file biomedicines-12-01874-s001.zip › Supplementary Figure/Figure S8/Leaveoneout plot of MR analysis for TL to ubm-b-2054.pdf]

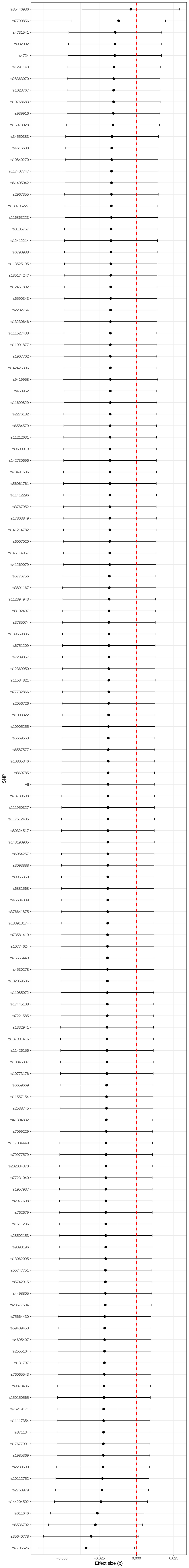
rs35446936
rs7790856
rs4731541
rs932002
rs4724
rs1291143
rs28363070
rs1023767
rs10768683
rs939916
rs16978028
rs34550383
rs4616688
rs10840270
rs117407747
rs61405042
rs2967355
rs139795227
rs116863223
rs8105767
rs12412214
rs6790988
rs113525195
rs185174247
rs12451892
rs6590343
rs2282764
rs13230646
rs111527438
rs11991877
rs1907702
rs142426306
rs9419958
rs450962
rs11699829
rs2276182
rs6584579
rs11212631
rs9600019
rs142730696
rs78491606
rs56061761
rs11412296
rs3767952
rs17803849
rs141214782
rs6007020
rs145114957
rs41269078
rs6776756
rs3891167
rs112394943
rs8102497
rs3785074
rs139669835
rs6751209
rs7209057
rs12369950
rs11584821
rs77732866
rs2056726
rs1003322
rs10905255
rs6669563
rs6587577
rs10805346
rs869785
All
rs73730598
rs111950327
rs117512405
rs80324517
rs143190905
rs6054257
rs3093888
rs9955360
rs6881568
rs45604339
rs376641875
rs188918174
rs73581419
rs10774624
rs76666449
rs4530278
rs182059586
rs11085072
rs17445108
rs7221585
rs1332941
rs137901416
rs11426156
rs10845387
rs10773176
rs6659669
rs11557154
rs2538745
rs41304832
rs7099229
rs117034449
rs79977579
rs202034370
rs77231040
rs1957937
rs2977608
rs762679
rs1611236
rs28502153
rs9398196
rs13062095
rs55747751
rs5742915
rs4498805
rs28577594
rs75664430
rs59409453
rs4695407
rs2555104
rs131797
rs76065543
rs9878436
rs150150565
rs76219171
rs11117354
rs871134
rs17677991
rs1985369
rs2230590
rs10112752
rs2763979
rs144204502
rs611646
rs6536702
rs35640778
rs7705526
SNP
-0.050
-0.025
0.000
0.025
Effect size (b)
